# Retraction: Antibiotics Threaten Wildlife: Circulating Quinolone Residues and Disease in Avian Scavengers

**DOI:** 10.1371/annotation/6f4d2406-f4f8-4771-a5f7-a781e5312f3f

**Published:** 2012-10-22

**Authors:** Jesús Á. Lemus, Guillermo Blanco, Javier Grande, Bernardo Arroyo, Marino García-Montijano, Felíx Martínez

The authors wish to retract the PLOS ONE article ”Antibiotics threaten wildlife: circulating quinolone residues and disease in avian scavengers”.

The Ethics Committee of the Spanish Superior Council of Scientific Research (CSIC) has carried out a formal investigation in relation to concerns about potential scientific misconduct by Jesús A. Lemus, one of the authors of this article. The investigation has questioned the validity of the laboratory analyses conducted by Dr. Lemus; concerns were also raised about the existence of co-author Javier Grande.

Given the ephemeral nature of the material used (fresh plasma, swabs and tissues), we are unable to repeat these analyses with the same samples in order to verify the presence of veterinary drugs and pathogens. 

We sincerely apologize for any inconvenience to the readership of PLOS ONE.

